# Evaluation of the Saponin Content in *Panax vietnamensis* Acclimatized to Lam Dong Province by HPLC–UV/CAD

**DOI:** 10.3390/molecules26175373

**Published:** 2021-09-03

**Authors:** Huy Truong Nguyen, Kim Long Vu-Huynh, Hien Minh Nguyen, Huong Thuy Le, Thi Hong Van Le, Jeong Hill Park, Minh Duc Nguyen

**Affiliations:** 1 Faculty of Pharmacy, Ton Duc Thang University, Ho Chi Minh City 700000, Vietnam; nguyentruonghuy@tdtu.edu.vn (H.T.N.); vuhuynhkimlong@tdtu.edu.vn (K.L.V.-H.); nguyenminhhien@tdtu.edu.vn (H.M.N.); lethuyhuong@tdtu.edu.vn (H.T.L.); 2 Faculty of Pharmacy, University of Medicine and Pharmacy at Ho Chi Minh City, Ho Chi Minh City 700000, Vietnam; levan@uphcm.edu.vn; 3 College of Pharmacy, Seoul National University, Seoul 08826, Korea

**Keywords:** *Panax vietnamensis*, Vietnamese ginseng, HPLC–CAD, majonoside, vina-ginsenoside, ocotillol, Lam Dong, acclimatization

## Abstract

*Panax vietnamensis*, or Vietnamese ginseng (VG), an endemic *Panax* species in Vietnam, possesses a unique saponin profile and interesting biological activities. This plant is presently in danger of extinction due to over-exploitation, resulting in many preservation efforts towards the geographical acclimatization of VG. Yet, no information on the saponin content of the acclimatized VG, an important quality indicator, is available. Here, we analyzed the saponin content in the underground parts of two- to five-year-old VG plants acclimatized to Lam Dong province. Nine characteristic saponins, including notoginsenoside-R1, ginsenoside-Rg1, -Rb1, -Rd, majonoside-R1, -R2 vina-ginsenoside-R2, -R11, and pseudoginsenoside-RT4, were simultaneously determined by HPLC coupled with UV and with a charged aerosol detector (CAD). Analyzing the results illustrated that the detection of characteristic ocotillol-type saponins in VG by CAD presented a superior capacity compared with that of UV, thus implying a preferential choice of CAD for the analysis of VG. The quantitative results indicating the saponin content in the underground parts of VG showed an increasing tendency from two to five years old, with the root and the rhizome exhibiting different saponin accumulation patterns. This is the first study that reveals the preliminary success of VG acclimatization and thereby encourages the continuing efforts to develop this valuable saponin-rich plant.

## 1. Introduction

**Figure 1:**
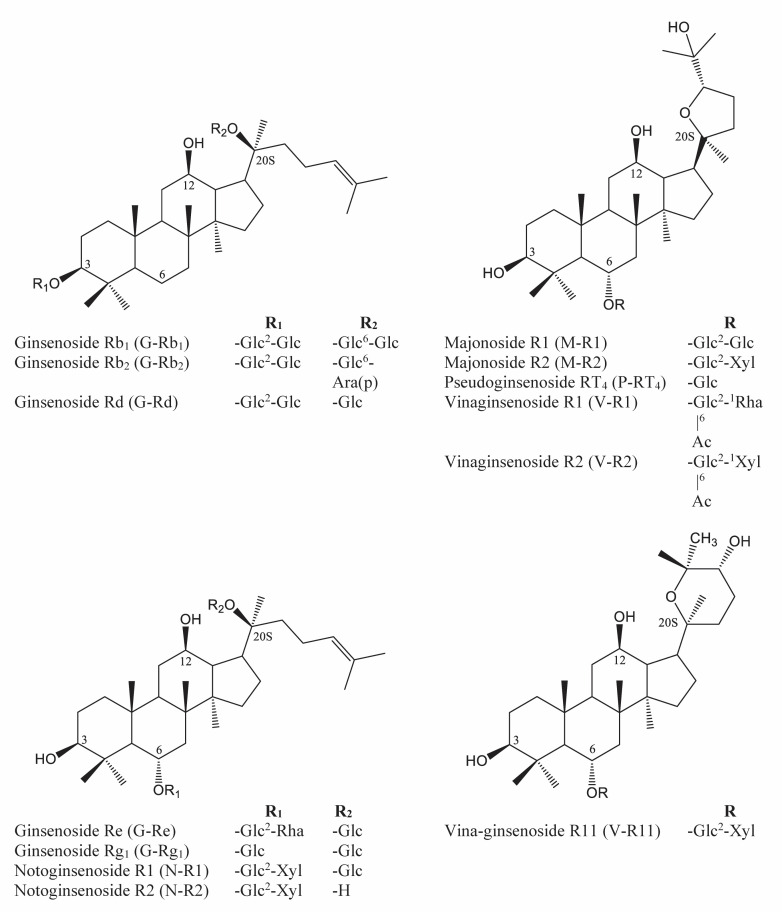
Structures of VG characteristic saponins.

*Panax vietnamensis* Ha et Grushv., commonly known as Vietnamese ginseng (VG), has been long used as a secret herbal medicine by the Sedang ethnic minority in Central Vietnam. Since its first discovery in 1973, VG has gained noticeably increasing interest from the scientific world. Many studies show that VG not only shares some similarities in protopanaxadiol-type saponins (PPD-type saponins) such as ginsenoside-Rb1 (G-Rb1), ginsenoside-Rc (G-Rc), ginsenoside-Rd (G-Rd), etc. and protopanaxatriol-type saponins (PPT-type saponins) such as ginsenoside -R1 (G-Rg1), ginsenoside -Re (G-Re), notoginsenoside-R1 (N-R1), etc., but also possesses a unique feature among the *Panax* genus due to its high content of ocotillol-type saponins (OCT-type saponins) such as majonoside-R1 (M-R1), majonoside-R2 (M-R2), vina-ginsenoside-R2 (V-R2), vina-ginsenoside-R11 (V-R11), pseudoginsenoside-RT4 (p-RT4), etc. (Figure 1). In fact, OCT-type saponins account for over 50% of the total saponin content in VG [1,2,3]. Especially M-R2, a major compound with a yield of over 5%, was proven to have promising biological effects, such as anti-tumor activity in a two-stage carcinogenesis test in mice models [4], protective activity against free radical-induced tissue injury in vitro [5], suppressive effects on psychological stress in mice models, etc. [6,7,8]. Concerning therapeutic value, VG is also proven to possess many interesting biological activities such as being anti-melanogenic, anti-oxidative, anti-stress, anti-cancer, hepato-cytoprotective, and having nephroprotective effects against cisplatin toxicity [9,10,11].

Due to its unique chemical profiles and beneficial therapeutic effects, VG has gained plenty of interest from consumers. Hence, to meet market demand, the wild VG has been increasingly exploited to the point of extinction. As a result, many efforts in acclimatizing VG to other suitable areas besides its original habitat have been carried out to preserve and develop this valuable *Panax* species. Yet, it is expected that due to the dissimilarities in cultivation areas, the saponin content of VG would vary as well, like other *Panax* spp. For example, Samukawa et al. reported that one factor resulting in changes to ginsenoside content in *Panax japonicus* (Japanese ginseng), including lateral roots, roots and rhizomes, was due to differences in the cultivation location, among others [12]. Wang et al. figured out the association of the major ginsenoside content variation in *Panax quinquefolius* at different ages with growing areas [13]. These reports suggested that VG cultivated in dissimilar regions would yield different saponin contents and accumulation patterns. However, there has been almost no information on saponin accumulation profiles of acclimatized VG, despite such information essentially serving as a valuable indication of the success of acclimatization.

So far, HPLC is considered a common separation and analysis method for the quantitative determination of the saponin content in *Panax* species due to its superior reliability, simplicity, and accuracy. For detection of eluted compounds from HPLC, ultraviolet detector (UV), evaporative light scattering detector (ELSD), charged aerosol detector (CAD), or mass spectrometer detector (MSD) are the most popular detectors for such analysis [14,15,16,17,18]. Among those, UV is widely considered the simplest, most robust, and most economical detection method. For PPD and PPT-type saponins, UV detection at low wavelengths (<205 nm) has been widely employed. However, as shown in Figure 1, it would be greatly challenged when detecting OCT-type saponins due to the lack of chromophore in their structures, even at a very low wavelength (<198 nm). In contrast to UV detection, CAD, a relatively new universal HPLC detector, could well detect these chromophore-free compounds. The mechanism of CAD is that a corona discharge needle charges the dried particle stream. Afterward, an electrometer measures the resulting electrical charge, resulting in the universal detection of compounds [19]. With the strength of a universal detector, CAD has proven its efficacy in determining ginsenosides in *Panax* species, with good linearity and precision [20].

In this study, we collected the underground part of two- to five-year-old VG plants acclimatized to Lam Dong, a highland province more southward from the natural habitat of VG. The content of nine characteristic saponins in VG roots and rhizomes at different growth ages was then analyzed by HPLC coupled with UV and CAD. The result from two detectors implied the superior detection capacity of CAD when it comes to detecting the characteristic ocotillol-type saponins, while the quantitative analysis indicated that the saponin content in the underground parts of VG showed an increasing tendency with age, with the root and the rhizome possessing different accumulation patterns.

## 2. Results and Discussion

### 2.1. Assessing the Quantitative Performance of UV and CAD

Nine saponins, including N-R1, M-R1, G-Rg1, M-R2, V-R11, p-RT4, V-R2, G-Rb1, and G-Rd in the sample extract, were separated by RP-HPLC and detected by UV and CAD. The regression relationship between the analyte concentration and measured signal for nine saponins is listed in Table 1. The linearity of the regression curves was assessed by the correlation coefficients. The precision of injection was retrieved by analyzing the peak area variations in six injections of standard solutions, while the intra-day (triplicate extraction on a single day) and inter-day (three different days) precisions are 0.09–1.4% (*n* = 3) and 2.37–3.03% (*n* = 3), respectively. The recoveries of the saponins were determined with spiked samples. Nine saponin standards including N-R1 (0.210 mg/mL), M-R1 (0.500 mg/mL), G-Rg1 (0.620 mg/mL), M-R2 (0.620 mg/mL), V-R11 (0.330 mg/mL), p-RT4 (0.560 mg/mL), V-R2 (0.500 mg/mL), G-Rb1 (0.390 mg/mL), and G-Rd (0.580 mg/mL) were added into 100 mg of four-year-old VG root and extracted with the described method. HPLC–CAD showed good recoveries of nine saponins within the range of 90–111.5% (*n* = 3).

**Figure 2:**
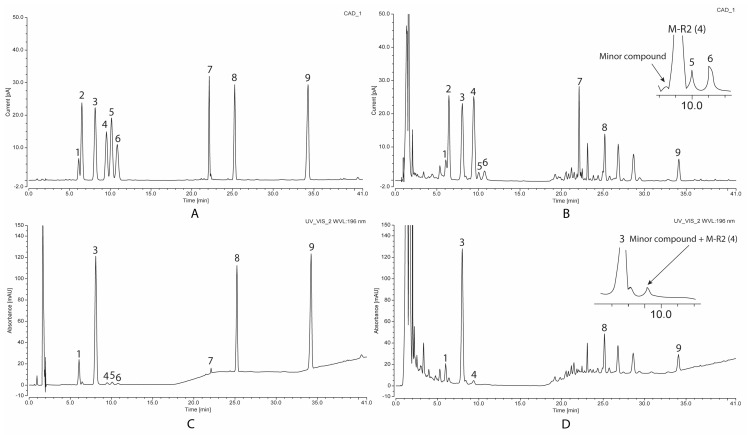
Representative chromatogram of standard mixture detected by CAD (**A**) and UV (**C**), and VG extract detected by CAD (**B**) and UV (**D**). **1**: N-R1; **2**: M-R1; **3**: G-Rg1; **4**: M-R2; **5**: V-R11; **6**: p-RT4; **7**: V-R2; **8**: G-Rb1; **9**: G-Rd.

As mentioned above, VG contains an abundance of OCT-type saponins without double bonds in their structures. Therefore, the detection of this type of saponin in VG extract by UV is a challenge, and a very low wavelength (<198 nm) must be used to analyze these saponins. Although the content of M-R2, the main OCT-type saponin of VG, is relatively higher than that of G-Rg1 and G-Rb1, the two other VG characteristic PPT- and PPD-type saponins, the LOD and LOQ of M-R2 is 0.0312 mg/mL and 0.0945 mg/mL, respectively, which is 13 times higher than those of G-Rg1 and 23–24 times higher than those of G-Rb1. Moreover, in Figure 2, the detection at such a low wavelength also caused a baseline shift due to the change in mobile phase composition. These nine saponins of interest have different polarities ranging from high polarity (N-R1, M-R1, G-Rg1, M-R2) to medium polarity (V-R2, G-Rb1) to lower polarity (G-Rd). Therefore, a gradient elution should be applied with acetonitrile concentration increasing from 21% to 40%. As a result, a baseline shift with high noise was obtained. The detection at a low wavelength could also detect many minor compounds, which leads to confusion in the detection and quantitative determination of M-R2 and V-R2. The zoomed part in chromatography in Figure 1 shows a minor compound eluting just before M-R2, which might overlap the peak of M-R2 when there is an unexpected minor change of analysis condition. As a result, the peak of M-R2 may broaden, hence possibly increasing the peak area parallel with the increase in the content of this key compound. The same problem also occurred to V-R2, where many co-eluted minor compounds make V-R2 undetectable. To deal with this problem, the weaker mobile phase needs to be used to separate M-R2 or V-R2 from other minor compounds. This resulted in a significant increase in the retention time, broadening peaks, and a significant increase in the LOD and LOQ value. In addition, other lower content ocotillol-type saponins such as M-R1, V-R11, and p-RT4 were unable to be detected. Without the data of M-R1, V-R2, V-R11, and p-RT4 content, the estimation of total saponin content in VG extract by the sum of all content of detected saponins is not reliable. Thus, the HPLC–UV method posed great difficulty in analyzing the characteristic ocotillol-type saponins of VG.

Next, we evaluate the performance of CAD on the detection and quantitative determination of saponins. With the strength of a universal detector, CAD was expected to overcome all the problems of the UV detector. Regarding the sensitivity in detecting OCT saponins, the LOD and LOQ of M-R2 are 0.0076 mg/mL and 0.0229 mg/mL, respectively, which is four times lower than that of M-R2 detected by UV. CAD could also well detect V-R2, another marker saponin of VG with LOD of 0.0064 mg/mL and LOQ of 0.0195 mg/mL. With such high sensitivity, CAD could quantitatively and effectively determine OCT-type saponins in two- or three-year-old VG in low content. Another advantage of CAD is that the detection is not affected by the change of mobile phase ratio due to the evaporating step to remove the mobile phase before the elution entering the detection part. In the CAD chromatogram of VG extract, the abundance of M-R2 and V-R2 peaks was significantly higher than surrounding minor compounds. As a result, these minor compounds’ overlapping peaks caused no remarkable change in quantitative results. Therefore, in this particular case, CAD is much more robust than UV in detecting OCT-type saponins. Thanks to the strength of a universal detector, the total saponin content of VG extract could be estimated with high reliability. With these mentioned advantages of CAD in detecting characteristic saponins in VG, HPLC coupled with CAD was chosen to analyze the saponin contents in VG extracts.

### 2.2. The Change in Weight of Underground Parts of VG with Age

**Figure 3:**
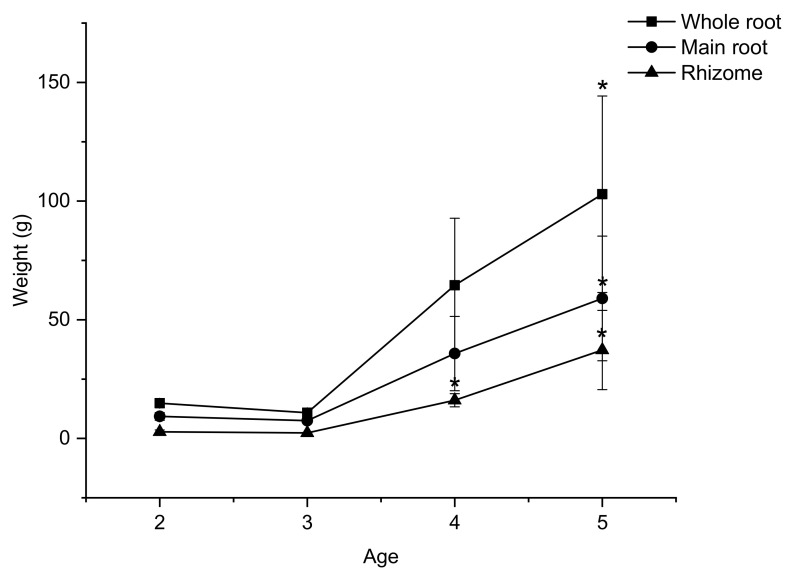
The increase trend weight of VG whole roots, main roots, and rhizomes from 2–5 years. Results are expressed as mean ± SD (*n* = 5). * *p* < 0.05 compared with the age of 2.

As shown in Figure 3, the weight of underground parts, including VG’s main root and rhizome, remains unchanged from two to three years and then increases gradually until five years of age. From two to five years, the weight of the main root increased only 6.34 times, while the weight of the rhizome increased 13.47 times. This result illustrates that the accumulation rate of mass in the rhizome is about two times higher than that of the main root. Yet, the main root still accounts for 61.28% of the weight of the whole underground part. Thus, the total saponin content of the main root will dictate the total saponin content of the whole root.

Interestingly, previous research on VG cultivated in Tra Linh indicated the reverse pattern in the accumulation rate of mass, with the accumulation rate of the main root being four times higher than that of the rhizome [21]. This result illustrates that the differences in cultivation area might affect the accumulation rate of mass in VG.

### 2.3. The Saponin Content of VG Main Roots at Different Ages

**Figure 4:**
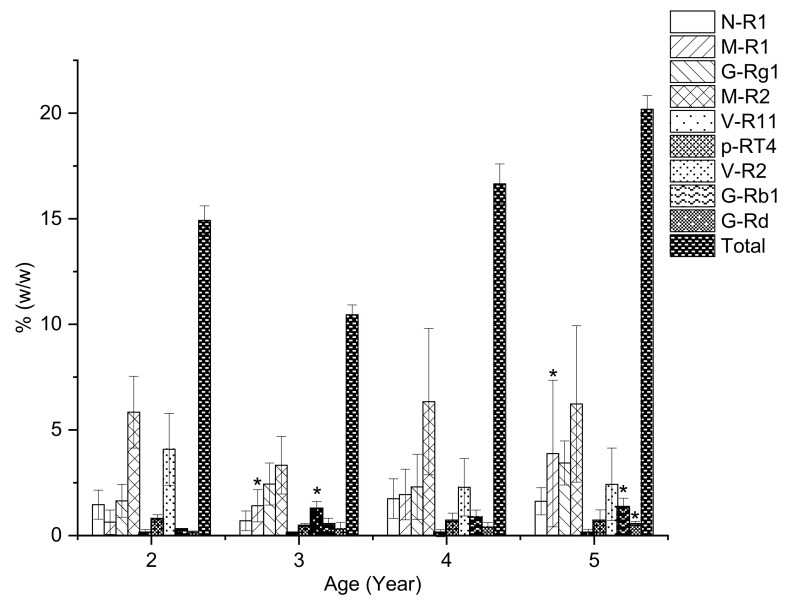
The change of saponin content in 2–5 years old VG main roots analyzed by HPLC–CAD. Results are expressed as mean ± SD (*n* = 5). * *p* < 0.05 compared with the age of 2.

The extracts of the main roots of two- to five-year-old VG were analyzed by HPLC–CAD and the changes of saponins could be observed in Figure 4. From two to three years of growth, the total saponin content fluctuated (10.5–14.9%), and then increased quickly to 16.7% at the age of four, ultimately reaching 20.2% at five years of growth. M-R1, G-Rg1, and G-Rb1 are the major saponins that mainly contribute to the increase in total saponin. From two to five years, M-R1 gradually increased from 0.63% at 2 to 1.41% at 3. At four years old M-R1 content was 1.94%, and then it increased quickly to 3.88% at five. At the age of five, the content of M-R1 was six times higher than that of the two-year-old VG root. G-Rb1 is a PPD-type saponin that shares a similar increasing pattern with M-R1. The G-Rb1 content was 0.24%, 0.51%, 0.86%, and 1.30% at two, three, four, and five years old, respectively. The content of this PPD-type saponin at five years old is 5.3 times higher than that of the first two years of growth. However, the increase pattern of G-Rg1 is quite different from M-R1. From two to three years old, the content of this PPT-type saponin increased quickly from 1.64% to 2.44%, then stayed stable during the next year of growth before increasing quickly to 3.44%. In contrast, M-R2, the major saponin of VG root, stayed stable during five years of growth with an average content of 5.43%. Surprisingly, the content of V-R2 was the highest at the age of two (4.05%) and gradually decreased to 1.30% at five. The summarized quantitative results of main roots are shown in Table 2, while detailed information can be found in Table S1.

### 2.4. The Saponin Content of VG Rhizomes at Different Ages

**Figure 5:**
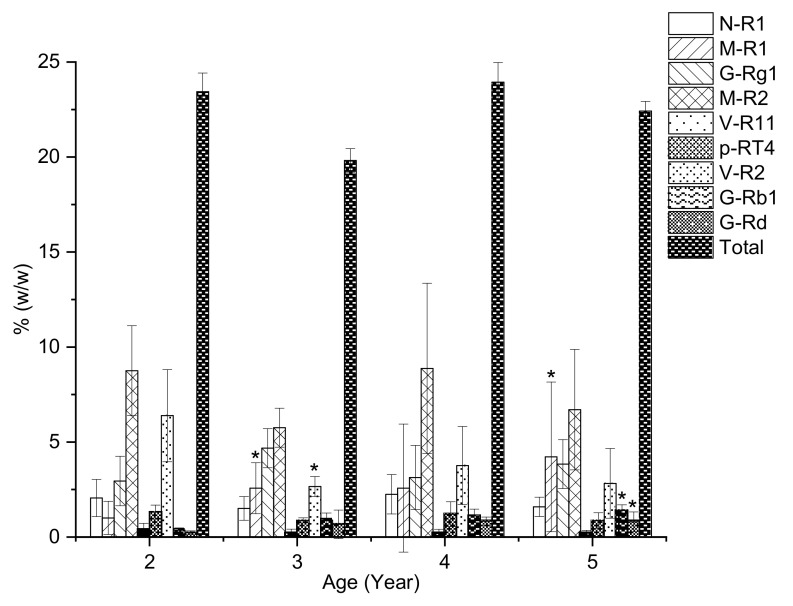
The change of saponin content in 2–5 years old VG rhizomes analyzed by HPLC–CAD. Results are expressed as mean ± SD (*n* = 5). * *p* < 0.05 compared with the age of 2.

Unlike the main root, the total saponin content of rhizomes accumulated at a high level from two years old and stayed stable during five years of growth, as shown in Figure 5. The stability of total saponin content may be explained by the increase of M-R1, G-Rb1, and G-Rd content accompanied by the decrease of V-R2 content. In detail, from two to three years old, the content of M-R1 increased 2.6 times (1.00–2.57%) and then rose sharply to 4.22% at the age of five. Unlike M-R1, the content of G-Rb1 and G-Rd increased gradually during the time of growth. After five years of growth, the content of G-Rb1 increased 3.9 times (0.36–1.40%), while the increase rate of G-Rd is 3.4 times (0.24–0.80%). In contrast, the V-R2 content decreased gradually from 6.39% at two to 2.66% at three and then stayed unchanged. The content of other major saponins such as G-Rg1 and M-R2 stayed relatively stable during growth. The summarized quantitative results of rhizomes were shown in Table 3, while detailed information can be found in Table S2.

### 2.5. The Saponin Accumulation Pattern in Various Parts of VG at Different Ages

**Figure 6:**
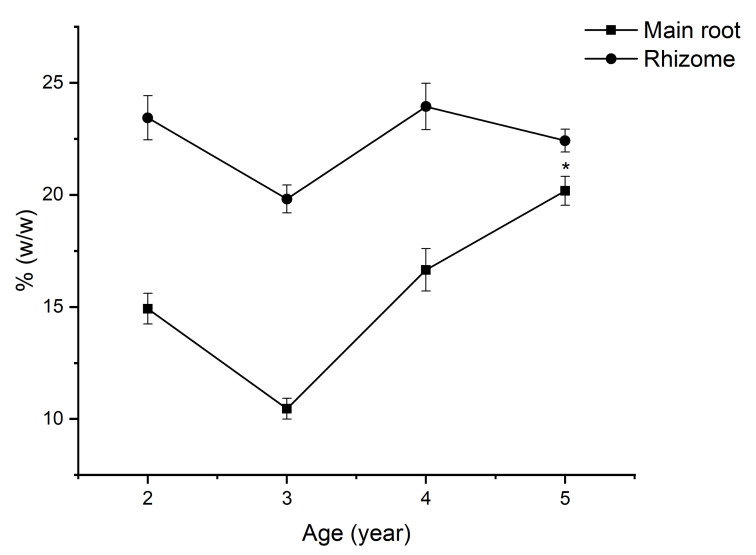
The saponin accumulation pattern in the main root and rhizome of VG. Results are expressed as mean ± SD (*n* = 5). * *p* < 0.05 compared with the age of 2.

The total saponin content in radix and rhizomes of VG in this study demonstrates two distinct saponin accumulation trends in Figure 6. In the rhizome, the saponin content accumulates highest at two years old, beginning the growth period. In contrast, in the root, the saponin accumulation peaked at the age of five. Yet, the total saponin content in radix and rhizome witnessed the same drop at the age of three, and OCT-type saponins such as V-R2, M-R2, and a PPT-type saponin N-R1 are the main causes for this drop in both parts.

In this case of VG cultivated in Lam Dong, the accumulation pattern of saponin in both root and rhizome are quite different from our previous report on VG cultivated in Tra Linh, in that it indicates that the total saponin content increases slightly from two to three years, then significantly from three to five years, and remains stable until seven years of growth. Additionally, it is worth noting that there is a significant difference in the content of M-R1 and V-R2 in VG cultivated in Lam Dong and VG cultivated in Tra Linh. For instance, at five years old, the content of M-R1 in the main root and rhizome of VG cultivated in Lam Dong was 3.88% and 4.22%, respectively, which is 25–35 times higher than those of the main root and rhizome of VG growth on Tra Linh Mountain. In addition, the content of V-R2 in the root and rhizome of VG in Lam Dong is 27 times and 25 times higher than the content of V-R2 in the root and rhizome of VG in Tra Linh, respectively [21]. This significant difference suggests that M-R1 and V-R2 could serve as a potential marker for distinguishing between VG cultivated in Lam Dong and Tra Linh. Therefore, future studies with a larger number of VG samples from these regions on the same HPLC system are expected to reconfirm this preliminary result.

## 3. Materials and Methods

### 3.1. General Experimental Procedures

HPLC analyses were performed on an Ultimate 3000 Thermo Fisher UHPLC system (Thermo Fisher Scientific, Waltham, MA, USA) equipped with a quaternary gradient pump, auto-sampler, a diode-array detector (DAD), and charged aerosol detector (CAD) connected to Chromeleon™ Chromatography Data System Software. A Luna C_18_ column (150 mm × 4.6 mm. i.d., 5 µm, Phenomenex, Torrance, CA, USA) was used to separate compounds of interest. HPLC-grade solvents were purchased from J.T.Baker (Deventer, The Netherlands).

### 3.2. Plant Material

The VG was harvested from Lam Dong province, Vietnam in December of 2019 and identified botanically by one of the authors (Prof. Minh Duc Nguyen). A voucher specimen sample of each age was deposited in the herbarium of the Faculty of Pharmacy, Ton Duc Thang University (TDTU-FoP-20190103, Ho Chi Minh City, Vietnam).

The fresh underground part of two- to five-year-old VGs (Figure S1, Supplementary Material) was harvested at the Viet Ginseng Corporation farm in Lam Dong Province (*n* = 5). Each group of age was collected directly from the corresponding cultivation bed. The rhizomes and main roots were cleaned and separated before being dried at 50 °C in an oven until fully dry. The dried samples were then ground and sieved to obtain less than 355 µm particle size powder.

Next, a representative sample of each group was qualitatively determined by usingthin-layer chromatography (TLC) according to method described in Vietnamese Pharmacopoeia V. The result conformed to the criteria of the *Panax vietnamensis* monograph for the TLC identification test, hence further confirming the identities of the samples (Figure S2, Supplementary Material) [22].

### 3.3. Sample and Standard Preparation

An aliquot of 100 mg of VG powder was placed into a 15 mL centrifuge tube containing 10 mL of 70% aqueous methanol. The tubes were tightly capped, shaken well, and ultra-sonicated for 40 min at room temperature. During the extraction, the tubes were also shaken by vortex every 10 min. Afterward, the extract was centrifuged, and the supernatant was filtered through a 0.45 µm filter before being analyzed by HPLC–CAD.

G-Rb1, G-Rd, G-Rg1, N-R1, M-R1, M-R2, V-R2, V-R11, and p-RT4 reference standards were prepared in our laboratory. A mixed standard solution in methanol of nine saponins including N-R1 (0.265 mg/mL), M-R1 (0.565 mg/mL), G-Rg1 (0.620 mg/mL), M-R2 (0.620 mg/mL), V-R11 (0.335 mg/mL), p-RT4 (0.567 mg/mL), V-R2 (0.500 mg/mL), G-Rb1 (0.490 mg/mL), and G-Rd (0.680 mg/mL) was prepared. This mixed standard stock solution was then diluted to generate a series of six standard operating solutions of different concentrations.

### 3.4. HPLC Analysis

The separation of saponins in VG extract was achieved by using a Phenomenex Luna C_18_ column (150 mm × 4.6 mm. i.d., 5 µm) eluted by a mobile phase consisting of water (A) and acetonitrile (B) with the following gradient program: 0–11 min, 21% B; 11–25 min, 21–32% B; 25–35 min, 32–40% B; 35–40 min, 40–95% B; 40–60, 95% B; 60–61, 95–21% B; 61–71, 21% B. The flow rate was set at 1.0 mL/min, and the injection volume was 20 µL. The column temperature was maintained at 30 °C. The CAD detector was performed at the default manufacturing setting, while the UV detector was set at 196 nm.

Peak identities were indicated by comparing peak retention time to the retention time of corresponding standards. The quantitative determination of saponins was carried out using the linear calibration curve (UV) and the logarithm regression (CAD) established by the analysis of the standards series (Figure S3, Supplementary Material).

### 3.5. Statistical Analysis

The data are expressed as the mean ± standard deviation. The ANOVA followed by Dunnett’s post-hoc test was used with SPSS version 22.0 to compare the two age groups’ weight and content of saponins. A *p*-value of < 0.05 was considered statistically significant.

## 4. Conclusions

This study evaluated nine characteristic saponins in different parts of two- to five-year-old VGs, cultivated in Lam Dong, using HPLC–UV/CAD. The result illustrated that a VG’s main root and rhizome acclimatized to Lam Dong province possessed different saponin accumulation patterns. The total saponin content of the underground part showed an increasing tendency from two to five years old. Among nine saponins, M-R1 significantly increased in both main roots and rhizomes during five years of growth, while M-R2 remained stable as the most abundant compound in the VG’s underground part. The age of five witnessed the peak in both root weight and saponin accumulation. Additionally, apparent advantages of CAD compared with UV in the analysis of the characteristic saponins of VG were pointed out and discussed. Consequently, our study demonstrated that HPLC–CAD is superior to HPLC–UV as an effective and accurate method for quantitative and quantitative analysis of VG saponins. It also marks a preliminary success in acclimatizing the valuable VG to Lam Dong province in its total saponin content.

## Figures and Tables

**Table 1:** Calibration curve, concentration range, LOD, and LOQ of nine saponins.

Compounds	Calibration Curve	R^2^	Range (mg/mL)	LOD (mg/mL)	LOQ (mg/mL)
**UV**					
N-R1	y = 31.0544x + 0.095	0.9997	0.008–0.53	0.0024	0.0073
M-R1	N.D.	N.D.	N.D.	N.D.	N.D.
G-Rg1	y = 89.4402x + 0.2917	0.9999	0.019–1.24	0.0024	0.0073
M-R2	y = 1.4089x + −0.0086	0.9965	0.238–1.24	0.0312	0.0945
V-R11	N.D.	N.D.	N.D.	N.D.	N.D.
p-RT4	N.D.	N.D.	N.D.	N.D.	N.D.
V-R2	N.D.	N.D.	N.D.	N.D.	N.D.
G-Rb1	y = 66.7414x + 0.1563	1.0000	0.015–0.98	0.0013	0.0041
G-Rd	y = 71.789x + 0.2697	1.0000	0.21–1.36	0.0021	0.0065
**CAD**					
N-R1	log(y) = 0.8795 × log(x) + 0.8698	0.9983	0.008–0.53	0.0053	0.0159
M-R1	log(y) = 0.8053 × log(x) + 1.1228	0.9985	0.018–1.13	0.0092	0.0278
G-Rg1	log(y) = 0.8136 × log(x) + 1.1613	0.9981	0.019–1.24	0.0093	0.0281
M-R2	log(y) = 0.8565 × log(x) + 1.0409	0.9975	0.019–1.24	0.0076	0.0229
V-R11	log(y) = 0.8254 × log(x) + 1.3789	0.9961	0.010–0.67	0.0045	0.0138
p-RT4	log(y) = 0.8649 × log(x) + 1.0157	0.9970	0.018–1.13	0.0071	0.0214
V-R2	log(y) = 0.7981 × log(x) + 1.0384	0.9970	0.016–1.00	0.0064	0.0195
G-Rb1	log(y) = 0.8161 × log(x) + 1.2291	0.9983	0.015–0.98	0.0078	0.0236
G-Rd	log(y) = 0.8165 × log(x) + 1.2531	0.9987	0.21–1.36	0.0079	0.0240

**Table 2:** The saponin content in main roots of 2- to 5-year-old VGs detected by HPLC–CAD. Results are illustrated as mean ± standard deviation (*n* = 5). * *p* < 0.05 compared with the age of 2.

Age	Saponin Content (% *w*/*w*)									Total Saponin Content
	N-R1	M-R1	G-Rg1	M-R2	V-R11	p-RT4	V-R2	G-Rb1	G-Rd	
2	1.46 ± 0.69	0.63 ± 0.58	1.64 ± 0.78	5.84 ± 1.7	0.14 ± 0.14	0.75 ± 0.24	4.08 ± 1.7	0.24 ± 0.03	0.13 ± 0.09	14.92 ± 0.69
3	0.7 ± 0.47	1.41 ± 0.77 *	2.44 ± 1	3.33 ± 1.36	0.09 ± 0.06	0.41 ± 0.15	1.27 ± 0.34 *	0.51 ± 0.32	0.3 ± 0.33	10.46 ± 0.47
4	1.74 ± 0.94	1.94 ± 2.72	2.31 ± 1.54	6.34 ± 3.46	0.11 ± 0.17	0.67 ± 0.4	2.29 ± 1.35	0.86 ± 0.35	0.39 ± 0.23	16.65 ± 0.94
5	1.62 ± 0.64	3.88 ± 3.47 *	3.44 ± 1.04	6.23 ± 3.71	0.14 ± 0.16	0.67 ± 0.54	2.43 ± 1.72	1.3 ± 0.47 *	0.48 ± 0.19 *	20.18 ± 0.64

**Table 3:** The saponin content in rhizomes of 2–5 years old VGs detected by HPLC–CAD. Results are illustrated as mean ± standard deviation (*n* = 5). * *p* < 0.05 compared with the age of 2.

Age	Saponin Content (% *w*/*w*)									Total Saponin Content
	N-R1	M-R1	G-Rg1	M-R2	V-R11	p-RT4	V-R2	G-Rb1	G-Rd	
2	2.05 ± 0.98	1.01 ± 0.87	2.95 ± 1.3	8.76 ± 2.37	0.37 ± 0.35	1.32 ± 0.37	6.39 ± 2.42	0.36 ± 0.12	0.24 ± 0.09	23.44 ± 0.98
3	1.51 ± 0.62	2.57 ± 1.34 *	4.68 ± 1.03	5.75 ± 1.03	0.21 ± 0.21	0.88 ± 0.13	2.66 ± 0.52 *	0.89 ± 0.38	0.67 ± 0.75	19.82 ± 0.62
4	2.25 ± 1.04	2.58 ± 3.37	3.14 ± 1.69	8.88 ± 4.82	0.19 ± 0.22	1.21 ± 0.65	3.76 ± 2.06	1.12 ± 0.35	0.83 ± 0.22	23.94 ± 1.04
5	1.59 ± 0.51	4.22 ± 3.94 *	3.84 ± 1.29	6.7 ± 3.17	0.17 ± 0.19	0.87 ± 0.41	2.82 ± 1.83	1.4 ± 0.3 *	0.8 ± 0.52 *	22.42 ± 0.51

## Data Availability

Data is contained within the article or Supplementary Material.

## Supplementary Materials

The following are available online: Figure S1: Underground parts of 2–5 years old VG collected in Lam Dong, Vietnam, Figure S2: Identification of two- to five-year-old *Panax vietnamensis* acclimatized to Lam Dong by thin-layer chromatography compared with standards. S, Standard mixture, VG: *P. vietnamensis* standard material, 2–5: *P. vietnamensis* 2–5 years old cultivated in Lam Dong. Figure S3: Calibration curve of 9 saponins detected by CAD (a) and UV (b), Table S1. The saponin content in main roots of two- to five-year-old VGs detected by HPLC–CAD, Table S2. The saponin content in main roots of two- to five-year-old VGs detected by HPLC–CAD.

## Sample Availability

Samples of the two- to five-year-old *Panax vietnamensis* main root and rhizomes are available from the authors.
