# Supplementary material for: Evaluation of the Saponin Content in Panax vietnamensis Acclimatized to Lam Dong Province by HPLC–UV/CAD
Source: Molecules. 2021 Sep 3;26(17):5373. doi: 10.3390/molecules26175373 (PMC8433671; doi:10.3390/molecules26175373)
Supplement: Supplementary file 1 [file molecules-26-05373-s001.zip › molecules-1353964-supplementary.pdf]

molecules

MDPI

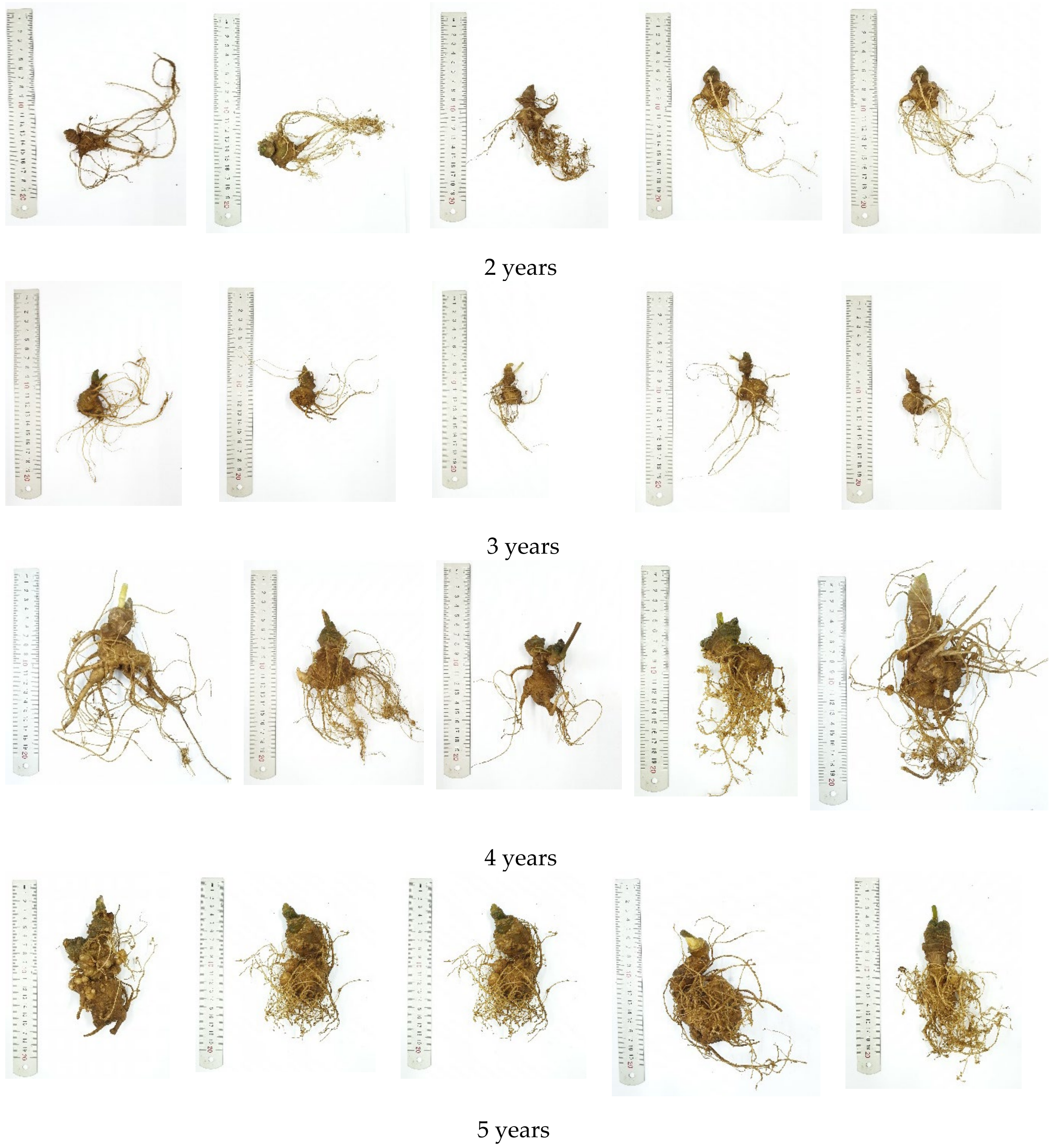

Figure. S1. Underground parts of 2–5 years old VG collected in Lam Dong, Vietnam.

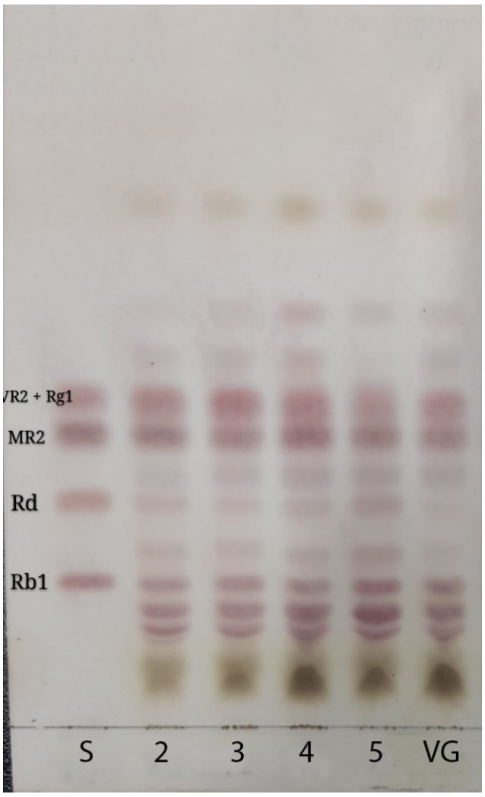

Figure S2. Identification of 2-5 years *Panax vietnamensis* acclimatized to Lam Dong by thin-layer chromatography compared with standards. S, Standard mixture, VG: P. vietnamensis standard material, 2-5: P. vietnamensis 2-5 years old cultivated in Lam Dong.

Plate: Silica gel (particle size 2-10 μM), Mobile phase: *n*-butanol – water – acetic acid (4:5:1, upper layer). Sample: To 0.1 g of powder, add 5 mL of 100% methanol, extract by sonication for 30 min, cool down and filter.

Standard references: mixture of ginsenoside-Rg1, ginsenoside-Rb1, majonoside-R2 in methanol (1 mg/mL). Reference drug: Prepare a solution of 0.1 g powder of P. vietnamensis reference drug in the same manner as the sample.

Detection: spray with 10% sulfuric acid solution in methanol, heat at 110 °C in 3 min, examine in daylight. The spots in the chromatograms obtained with the test solution correspond in position and color to the spots in chromatogram obtained with the reference drug and the reference solutions.

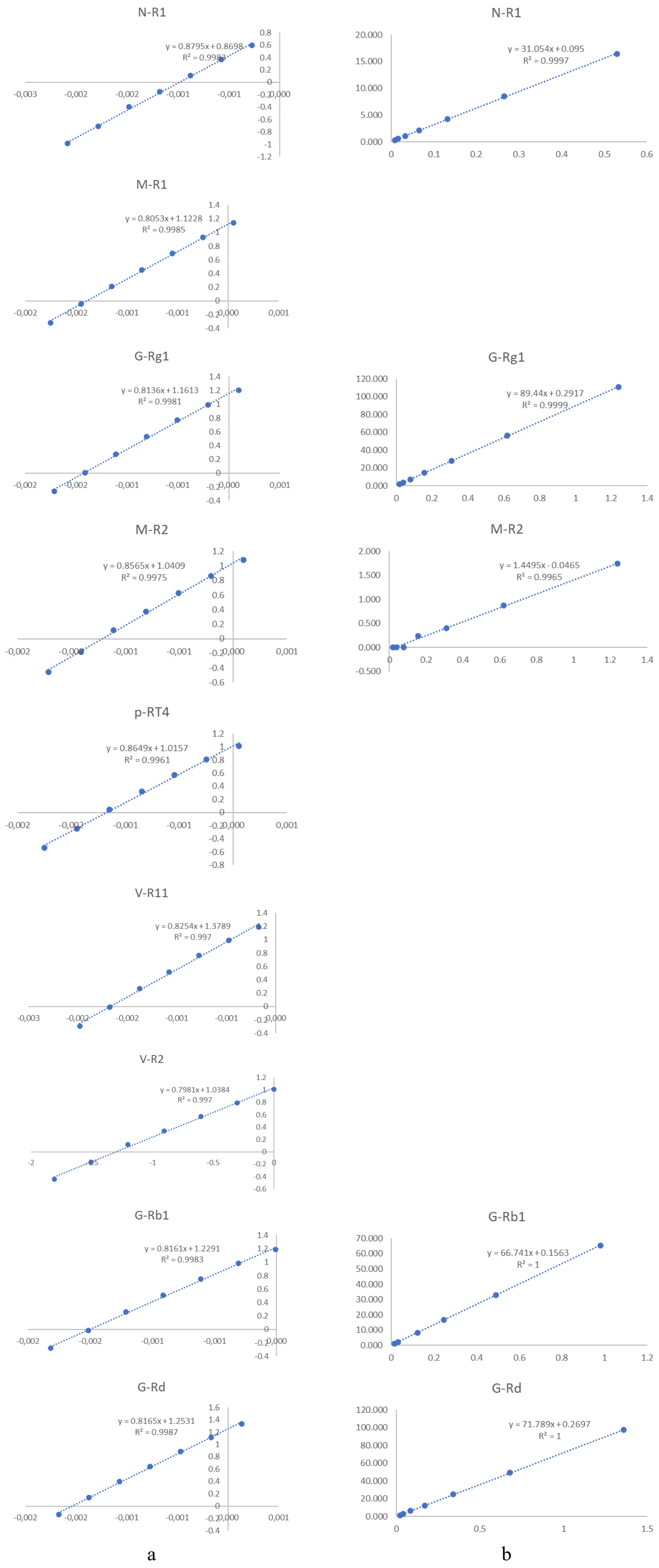

Figure S2. Calibration curve of 9 saponins detected by CAD (**a**) and UV (**b**).

Table S1. The saponin content in main roots of 2–5 year VGs detected by HPLC-CAD. Results are calculated as percentage of saponin weight per weight of dry samples. *$p < 0.05$ compared with that the age of 2

Age	Sample	Saponin content (% w/w)									Total saponin content
		NR1	MR1	Rg1	MR2	VR11	pRT4	VR2	Rb1	Rd	
2	1	2.185	0.271	1.301	6.761	0.028	0.966	3.947	0.280	0.102	15.840
	2	0.504	1.660	1.436	2.868	0.275	0.338	1.274	0.239	0.086	8.682
	3	1.925	0.376	1.981	6.971	0.053	0.847	4.354	0.204	0.185	16.896
	4	1.032	0.475	0.718	6.561	0.032	0.777	5.279	0.266	0.245	15.386
	5	1.664	0.388	2.783	6.048	0.314	0.807	5.551	0.230	0.015	17.800
	MEAN ± SD	1.46 ± 0.69	0.63 ± 0.58	1.64 ± 0.78	5.84 ± 1.7	0.14 ± 0.14	0.75 ± 0.24	4.08 ± 1.7	0.24 ± 0.03	0.13 ± 0.09	14.92 ± 0.69
3	1	0.957	0.210	2.016	4.076	0.039	0.426	1.232	0.343	0.085	9.384
	2	1.249	2.126	2.892	3.806	0.047	0.439	1.060	0.842	0.581	13.042
	3	0.295	1.516	1.895	2.002	0.158	0.408	1.416	0.178	0.057	7.926
	4	0.849	1.998	3.956	4.939	0.136	0.608	1.767	0.851	0.725	15.829
	5	0.138	1.215	1.441	1.817	0.052	0.183	0.870	0.314	0.061	6.092
	MEAN ± SD	0.7 ± 0.47	1.41 ± 0.77*	2.44 ± 1	3.33 ± 1.36	0.09 ± 0.06	0.41 ± 0.15	1.27 ± 0.34*	0.51 ± 0.32	0.3 ± 0.33	10.46 ± 0.47
4	1	1.560	0.158	0.670	7.021	0.032	0.609	2.449	0.501	0.254	13.254
	2	1.026	6.652	4.119	1.013	0.406	0.112	0.365	0.858	0.540	15.091
	3	1.018	1.945	2.291	5.261	0.020	0.541	1.564	0.947	0.172	13.758
	4	3.320	0.574	3.557	10.095	0.106	0.953	3.619	1.396	0.714	24.335
	5	1.791	0.385	0.892	8.312	0.000	1.130	3.435	0.619	0.264	16.828
	MEAN ± SD	1.74 ± 0.94	1.94 ± 2.72	2.31 ± 1.54	6.34 ± 3.46	0.11 ± 0.17	0.67 ± 0.4	2.29 ± 1.35	0.86 ± 0.35	0.39 ± 0.23	16.65 ± 0.94
5	1	2.084	3.539	3.321	11.590	0.136	1.481	4.151	1.461	0.298	28.061
	2	1.206	2.619	2.852	4.940	0.052	0.466	1.299	1.386	0.689	15.511
	3	2.530	0.374	3.320	7.683	0.018	0.877	4.349	1.935	0.619	21.705
	4	1.111	9.690	5.198	1.580	0.417	0.028	0.581	1.000	0.517	20.122
	5	1.190	3.197	2.496	5.347	0.055	0.501	1.751	0.704	0.280	15.521
	MEAN ± SD	1.62 ± 0.64	3.88 ± 3.47*	3.44 ± 1.04	6.23 ± 3.71	0.14 ± 0.16	0.67 ± 0.54	2.43 ± 1.72	1.3 ± 0.47*	0.48 ± 0.19*	20.18 ± 0.64

Table S2. Saponin content of VG rhizome from 2–5 year detected by HPLC-CAD. Results are calculated as percentage of saponin weight per weight of dry samples. *$p < 0.05$ compared with that the age of 2

Age	Sample	Saponin content (% w/w)									Total saponin content
		NR1	MR1	Rg1	MR2	VR11	pRT4	VR2	Rb1	Rd	
2	**1**	3.660	0.677	2.503	11.131	0.080	1.719	6.716	0.380	0.166	27.033
	2	1.075	2.568	3.331	4.932	0.514	0.719	2.331	0.456	0.275	16.201
	3	2.195	0.604	2.743	8.357	0.225	1.406	6.652	0.168	0.240	22.589
	4	1.614	0.620	1.300	9.317	0.123	1.324	7.494	0.459	0.359	22.611
	5	1.725	0.561	4.859	10.045	0.919	1.426	8.752	0.334	0.139	28.760
	MEAN ± SD	2.05 ± 0.98	1.01 ± 0.87	2.95 ± 1.3	8.76 ± 2.37	0.37 ± 0.35	1.32 ± 0.37	6.39 ± 2.42	0.36 ± 0.12	0.24 ± 0.09	2.05 ± 0.98
3	1	1.620	0.239	2.839	6.060	0.020	0.816	2.354	0.446	0.267	14.661
	2	2.128	3.600	5.156	6.452	0.083	0.838	1.994	1.390	1.632	23.272
	3	1.000	3.133	4.977	4.640	0.560	1.096	3.364	0.593	0.098	19.461
	4	2.057	2.711	5.166	6.903	0.167	0.882	2.883	1.038	1.325	23.132
	5	0.740	3.175	5.248	4.717	0.220	0.741	2.698	0.991	0.029	18.557
	MEAN ± SD	1.51 ± 0.62	2.57 ± 1.34*	4.68 ± 1.03	5.75 ± 1.03	0.21 ± 0.21	0.88 ± 0.13	2.66 ± 0.52*	0.89 ± 0.38	0.67 ± 0.75	19.82 ± 0.62
4	1	2.610	0.555	1.474	13.430	0.144	1.615	5.444	0.752	0.618	26.641
	2	0.973	8.330	5.288	1.325	0.577	0.316	0.642	1.144	1.136	19.730
	3	1.380	2.888	3.456	7.049	0.048	0.895	2.691	1.620	0.715	20.742
	4	3.430	0.536	4.065	11.499	0.094	1.187	4.836	1.252	0.989	27.890
	5	2.862	0.579	1.391	11.078	0.068	2.012	5.192	0.821	0.696	24.699
	MEAN ± SD	2.25 ± 1.04	2.58 ± 3.37	3.14 ± 1.69	8.88 ± 4.82	0.19 ± 0.22	1.21 ± 0.65	3.76 ± 2.06	1.12 ± 0.35	0.83 ± 0.22	23.94 ± 1.04
5	1	1.650	3.499	2.366	10.660	0.086	1.350	3.853	1.149	0.175	24.788
	2	1.144	2.869	3.863	6.171	0.130	0.745	1.748	1.836	1.443	19.948
	3	2.445	0.351	3.409	7.805	0.010	1.046	5.427	1.458	0.599	22.551
	4	1.353	10.873	5.902	1.907	0.497	0.245	0.783	1.471	1.230	24.262
	5	1.376	3.510	3.649	6.973	0.106	0.959	2.311	1.098	0.564	20.546
	MEAN ± SD	1.59 ± 0.51	4.22 ± 3.94*	3.84 ± 1.29	6.7 ± 3.17	0.17 ± 0.19	0.87 ± 0.41	2.82 ± 1.83	1.4 ± 0.3*	0.8 ± 0.52*	22.42 ± 0.51
